# Targeted Primary and Secondary Preventive Strategies for Depression among Malaysian Pharmacy Students

**DOI:** 10.3390/ijerph19159629

**Published:** 2022-08-05

**Authors:** Izyan A. Wahab, Khang Wen Goh, Zainol Akbar Zainal, Najlaa Siham Mohamed Yusof, Hasniza Zaman Huri, Sabrina Anne Jacob, Muhammad Najib Mohamad Alwi, Rosnani Hashim, Shairyzah Ahmad Hisham, Nurdiana Jamil

**Affiliations:** 1 Faculty of Pharmacy, University of Malaya, Kuala Lumpur 50603, Malaysia; 2 Faculty of Data Science and Information Technology, INTI International University, Nilai 71800, Malaysia; 3 Faculty of Pharmacy, University of Cyberjaya, Cyberjaya 63000, Malaysia; 4 Kurnia Primary Health Clinic, Kuantan 25150, Malaysia; 5 Strathclyde Institute of Pharmacy and Biomedical Sciences, University of Strathclyde, Glasgow G4 0RE, UK; 6 International Medical School, Management and Science University, Shah Alam 40100, Malaysia

**Keywords:** mental health, preventive strategies, university students, Malaysia

## Abstract

The global depression burden has remained a challenge throughout the pre- and post-pandemic era. The pandemic effect has led to the spiraling of mental disorders among young people who will be the next generation of leaders. This study aims to identify university students’ sociodemographic, psychosocial and academic backgrounds and performance associated with depression symptoms for the development of primary and secondary preventive strategies for mental health. A cross-sectional study was conducted using an online questionnaire distributed to 19 institutions in Malaysia offering a Bachelor of Pharmacy degree program. The self-rated Depression Anxiety Stress Scale (DASS-42) was used to assess depression symptoms. Pearson’s chi-square test and Fisher’s exact test were used to assess the investigated variables with depression symptoms. Independent T-test and one-way ANOVA were used to compare means of depression score across variables. Binary logistic regression was employed to examine the relationship between the investigated variables and depression symptoms. A total of 610 pharmacy students participated, of which 47% (n = 289/610) were having depression symptoms. Students who smoke nicotine and those who have separated parents, family history of mental illness, and poor academic performance were associated with depression symptoms (*p* < 0.05). Differences in geographical areas, race and religion also showed significant associations with depression symptoms. Parental marital status, poor academic performance, history of mental illness and comorbidities were statistically predicting depression symptoms (*p* < 0.05). Primary preventive strategies allowing students to harness healthy coping skills for stress, nicotine-free campaigns and a holistic curriculum are warranted. Secondary measures on mindfulness and compassion skills activities to benefit students who experienced early life crises are highly recommended. Enforcing these targeted strategies in collaboration with health and social sectors should be the primary agenda of universities to ensure their uptake.

## 1. Introduction

The 2019 Global Burden of Disease Study reported that depressive disorders are among the top ten causes of disabilities for people aged 10 to 24 years [1]. The situation became dire during the COVID-19 pandemic as an additional 53.2 million new depression cases were reported in 2020, and consistent with the pre-pandemic era, younger age groups were more affected than older age groups [2]. South Asian and low–middle-income countries have the highest percentage of depression cases [3,4], reflecting the need to urgently take action. In the latest update by the World Health Organization (WHO), the Comprehensive Mental Health Action Plan 2013–2030 incorporates broad indicators to measure progress on strategy implementation to promote and prevent mental health issues, including the existence of functioning multi-sectoral mental health programs at the national level [5]. Partnership with educational institutions is identified as an implementation option to undertake mental health research and to define and incorporate mental health components in undergraduate and postgraduate curricula [5]. Accordingly, collaboration with universities is viewed as important in the latest Malaysia National Strategic Plan for Mental Health 2020–2025, which has specifically imposed action to conduct research in populations at risk, aiming to identify predictors to better enhance facilities and strategies to curb depression among adolescents, women and the elderly population [6]. Current preventive strategies are largely focused on tertiary prevention strategies aiming to reduce the impact on individuals, families and communities instead of providing similar attention and resources to implement primary and secondary preventive strategies.

Psychological problems have become increasingly prevalent among university students. The prevalence of depression symptoms among university students in low- to middle-income countries was 24%; however, underestimation is expected because of the limited studies from low-income countries [4]. Psychological distress among university students starts as early as in first year university students because of environmental and psychosocial changes while coping with academic and social demands [7,8]. Pressure to pass exams and high expectations from universities and families are some of the sources of anxiety and depression [9]. Medical and health sciences students reported high levels of stress, leading to a low quality of life [10,11]. Risk factors of mental health issues in university students have previously been addressed in recent years [12,13,14,15,16], and several are highlighted by the WHO and national action plans in their general themes [5,6]. Nevertheless, the mental health of young adults continues to be disproportionally affected and has been intensified in the COVID-19 pandemic [3].

The physical distancing impact of the COVID-19 pandemic has unveiled many social aspects of students who are at high risk of developing mental illnesses. Students may have multiple roles and responsibilities at one time—as a student, a caretaker, a provider and as a son or daughter. Within these roles, socioeconomic status [17], sub-standard living environment [18], financial constraints and relationship instability [19] can contribute to depression development. The uncertain situation posed by the recent pandemic outbreak has caused fear and anxiety among students about insufficient knowledge and skills while in remote learning and career readiness and employment [20,21]. Concerns regarding fear of misunderstanding and stigma as well as confidentiality and inadequate mental health resources can pose barriers for university students to seek treatment [22,23].

The prevalence of depression among university students has remained a challenge at all stages of the pandemic—before, during and after. While universities around the world are diligently working for world university rankings, policies surrounding mental well-being for university students are limited, and resources to assist this development are restricted [24]. The impact of previous mental health policies on university students is uncertain, although efforts to increase awareness to seek help for mental issues have increased in recent years; nevertheless, improvements to access and timely intervention are warranted [25]. With the recent release of the revised national plan highlighting intra- and inter-sectoral collaboration, crisis preparedness and strengthening surveillance for early detection and timely intervention indicating targeted strategies for high-risk groups are urgently needed [6].

The interplay of sociodemographic, psychosocial and academic factors with depression symptoms has never been well explored for pharmacy students. The present study was conducted with a focus on specific characteristics of university students associated with depression symptoms for the consideration of primary and secondary preventive strategies by universities and other relevant stakeholders such as the health and social sectors.

Therefore, the objective of this study was to identify targeted strategies for primary and secondary prevention based on sociodemographic profiles, psychosocial elements and academic achievement to optimize university students’ mental health. Based on the review of the literature on the effects of various students’ profiles and characteristics on depression, we hypothesized that Malaysian pharmacy students with specific sociodemographic profiles, psychosocial elements and academic performances have higher values on the depression symptoms scale. These significant profiles are relevant for faculties and universities to strategically formulate primary and secondary preventive strategies in partnership with health and social sectors.

## 2. Materials and Methods

This study obtained approval from the University of Cyberjaya Research Ethics Review Committee (CUCMS/CRERC/ER/109). Due to the nature of this research, participants of this study did not agree for their data to be shared publicly, so supporting data are not available.

### 2.1. Source of Data

A cross-sectional observational study was conducted using a web-based questionnaire from August 2018 to September 2018. This questionnaire contained the study background, a consent form and the intended survey questions in an online Google form. All questions were multiple-choice questions or scales and were set as compulsory to answer. Survey submission did not occur unless the online survey form was completed. Students were sampled via purposive sampling method. Only consenting active students from undergraduate pharmacy programs at 19 institutions in Malaysia offering a Bachelor of Pharmacy (Hons.) degree were included. Students with a known history of clinical depression were identified and excluded from the survey if the survey participant answered ‘Yes’ to having been diagnosed with clinical depression. Based on information acquired from the institutions, there were 3000 active undergraduate pharmacy students from the 19 institutions. Representatives from each institution were approached via email and social media for the dissemination of the online questionnaire. The participating faculties of pharmacy were from Universiti Sains Malaysia, University of Malaya, Universiti Kebangsaan Malaysia, Universiti Teknologi MARA, Universiti Islam Antarabangsa, AIMST University, UCSI University, International Medical University, University of Nottingham Malaysia Campus, Cyberjaya University College of Medical Sciences, MAHSA University, SEGi University, Lincoln University College, Monash University Malaysia, Management and Science University, Taylor’s University, Asia Metropolitan University, University Kuala Lumpur—Royal College of Medicine Perak and KPJ Healthcare University College. In addition, the online questionnaire link was distributed to the pharmacy students via the Malaysian Pharmacy Students’ Association (MyPSA) official Facebook^®^ page. MyPSA is an official national pharmacy students’ association. Only approved MyPSA members are eligible to be in the MyPSA Facebook^®^ group. Assuming an overestimated response distribution of 50%, a minimum effective sample size of 341 was needed to achieve a confidence interval of 95% and a 5% margin of error.

### 2.2. Research Tool and Investigated Variables

The English version of the online questionnaire survey form consisted of 19 investigated variables and 14 self-rated depression questions from the original 42-item Depression Anxiety Stress Scale (DASS-42) [26]. DASS-42 is a set of self-measures for the three negative emotional states of depression, anxiety and stress, where each of these three states contains 14 questions [27]. Hence, only the 14 questions related to depression out of 42 overall were used in this study. DASS-42 has been shown to have excellent transcultural validity [27]. The investigated variables for sociodemographic, psychosocial and academic matters and their measures are summarized in Supplementary Table S1. The selection of these variables was from previous research on potential factors for depression development [12,13,14,15,16]. The students were asked to rate the depression symptoms they experienced over the past week using a 4-point Likert scale (0 = Did not apply to me at all; 1 = Applied to me to some degree, or some of the time; 2 = Applied to me to a considerable degree, or a good part of time; 3 = Applied to me very much, or most of the time). A total score with its classification was then assigned: (1) Normal (0–9), (2) Mild depression (10–13), (3) Moderate depression (14–20), (4) Severe depression (21–27) and (5) Very severe depression (28–42). The scoring was scaled on based on the DASS-42. Personal data such as name, contact number and address were not collected from the online questionnaire to ensure the privacy and reliability of the data. No reward or incentives were provided to the questionnaire participants.

A pilot study was conducted for 30 random students over a 2-week period to investigate face validity and reliability. All respondents rated the survey as suitable, relevant and able to be answered without difficulty, and the Cronbach’s alpha reliability coefficient was 0.97, which showed excellent reliability. Therefore, no modifications for the questionnaire were required.

### 2.3. Statistical Analysis

All analyses were conducted using the Statistical Package for Social Sciences (SPSS) version 23.0 for Windows. Baseline data were presented using descriptive statistics. The independent variables (sociodemographic, psychosocial and academic variables) were subjected to Pearson’s chi-square and Fisher’s exact tests with presence of depression symptoms as the dependent variable at a *p* < 0.05 level of significance. Meanwhile, the mean depression score was compared between various groups of the tested variables using one-way ANOVA and independent T-test. For post hoc analysis, the Bonferroni correction was employed, and the significance level was set at *p* < 0.003. In addition, the relationships between nicotine smoking, parental marital status, recent loss of someone, pharmacy as the 1st degree of choice, cGPA performance, family history of mental illness and comorbidity status and depression symptoms were analyzed using binary logistic regression at a <0.05 level of significance.

## 3. Results

A total of 610 pharmacy students responded to the survey (response rate of 20.3%, n = 610/3000). There was a preponderance of females (n = 512, 84%), and the majority were between 20 and 24 years old (n = 512, 84%), did not consume alcohol (n = 571, 93%), did not smoke (n = 602, 99%) or use recreational drugs (n = 604, 99%), lived at students’ hostels (n = 318, 52%) and were not married (n = 572, 94%). Respondents were mostly from public universities (n = 367, 60%), had no scholarship for the pharmacy degree program (n = 436, 72%) and had a good cGPA (n = 306, 50%), and one-third had a family monthly income between MYR 2000 (USD 482) and 5000 (USD 1204) (Table 1).

Among the 610 students, nearly half of them (n = 289, 47.4%) were found to have depression symptoms. Of these, 14.1% had mild depression symptoms, 18.9% had moderate depression symptoms, 7.7% had severe depression symptoms and 6.7% had very severe depression symptoms. Based on the 19 tested variables, smoking, separated parents, recent loss of someone close, pharmacy degree as first choice degree, cGPA status, having family history of mental illness and comorbidities were significantly associated with depression symptoms (Table 2). The full list of 19 variables is available in Supplementary Table S2.

The results are consistent with the significant higher mean depression scores in students who smoke nicotine, with divorced parents, with recent loss of someone close and with family members having mental health problems (Table 3). In addition, students whose pharmacy degree was not their first-choice degree and those with poor academic performance similarly had higher depression scores. It is worth noting that students aged between 25 and 29 years (professional year) had statistically significant higher depression scores compared to students of other age groups. Students from Sabah and Sarawak (labeled as Others) had the highest depression score, followed by Indian, Malay and Chinese students living in Peninsular Malaysia. Among the different religions, students of other religiosity were found to score the highest mean depression score compared to Islamic, Buddhist, Hindu and Christian religions. Higher depression scores were also seen in those with a low family income and regular use of recreational drugs. The full list of the 19 variables and the mean depression scores is available in Supplementary Table S3.

The binary logistic regression identified divorced or separated parental marital status, poor cGPA, having family history of mental illness and underlying comorbidities as significantly predicting depression symptoms (Table 4). Smoking, recent loss of someone close and pharmacy degree as the first choice could not significantly predict development of depression symptoms.

## 4. Discussion

This study provides insights on various sociodemographic, psychosocial and academic factors that may contribute to depression development among undergraduate pharmacy students. Four specific characteristics significantly predicted depression symptoms: parental marital status, academic achievement, family history of mental illness and comorbidities (Table 4). These predictors are changeable, and every effort to identify and implement depression prevention programs should be made to avoid future harm and potentially help to reduce healthcare costs. There were no existing studies on pharmacy students that directly investigated parental marriage status, academic performance status, family history of mental illnesses and comorbidities as factors contributing to depression. However, other proxies such as family distress [13] and academic stress [14] were found to be significantly associated with depression. Studies on other higher degree programs showed similar findings where parental divorce [15], family history of mental illnesses [28] and poor health [16] significantly affect students’ mental health. The present study used specific status indicators rather than subjective proxies to strategically plan preventive strategies. Even though nicotine smoking did not significantly predict depression symptoms in the present study, previous studies have illustrated that smoking is common in young adults and people with mental health issues [29,30,31] and is a risk factor for mental illness among students [32]. In addition, premature death was associated with people with mental illness who had a longer history of tobacco use [33].

Primary preventive strategies reflect measures to prevent the onset of depression symptoms leading to its final diagnosis. The findings of this study show that primary preventive strategies should focus on implementing nicotine-free campaigns more aggressively and carefully design curricula that empower students to pursue a career pathway. The primary reason to start smoking among Turkish and Spanish university students was to relax and as a coping strategy to reduce stress [34,35]. Smoking abstinence has been proven to improve serious mental illness [36]. Smoke-free policies and campaigns have been successfully implemented at several universities in developed countries, which had positive impacts on behavior [37,38]. In addition, regular physical activities have been associated with better mental health and stress relief, which can be used by universities concurrently with smoke-free campaigns [39]. Several countries have proposed a smoking generation ban, including Malaysia and New Zealand, for those born after 2005 [40] and 2008 [41], respectively, seeing the urgency to curb the hazard impact from smoking.

Secondly, a primary preventive strategy for mental health can be incorporated into academic curriculum design, delivery and assessment methods, where it should be carefully strategized to avoid overburden of assignments and examination. This study found significantly higher mean depression scores among students with poor academic performance, which significantly predicted depression symptoms. Academic stressors such as frequent examinations and multiple assignments can partly contribute to depression among pharmacy students [14]. Reduced concentration and loss of interest and energy are some of the common depression symptoms among students. Identifying the areas of study in which students are struggling and failing can assist with developing appropriate remediation strategies and creating a non-stigmatized academic environment. Promoting a rewarding feeling or sense of satisfaction towards a profession during teaching and learning activities is another active coping strategy for stress when designing a curriculum [34].

This study also highlights the importance of own choice or decision to pursue a degree of interest. Students who selected pharmacy as their first choice were less depressed than students who did not consider pharmacy as their first choice. Choice ranking is therefore important and needs to be addressed during the interview session during students’ recruitment. Similarly, in another health education area, nursing students who made family-based decisions regarding their career path were found to be more depressed [42]. Medical students were also more burned out and depressed due to family pressure to become doctors to gain higher social status [43].

Satisfaction from making a decision without constraint or necessity is an important factor of well-being for potential university students and their family members. The sense of fulfillment from the student’s own choice can be reflected by the subsequent smooth transition to university life. In addition, the contentment from own choice inadvertently causes students to be aware of their own responsibility, which increases the chances for academic achievement and persistency towards their career pathway. Clear educational goals and career pathways surrounded with a supportive environment are important to be exposed to in the first year and maintained throughout the undergraduate program. Mindfulness and compassion skills introduced as early as in the first year of university life were hypothesized to be able to enhance preparedness to cope, productive stress responses and healthy post-coping reflections [44]. Sense of belonging to a university committee is an important area to be considered to curb stress among new university students [45]. Team-based learning, experiential learning and inter-professional learning as early as in the first year of university can increase students’ retention and satisfaction towards their degree program [46,47]. These academically constructive yet social activities planned for students can be another approach for healthy coping strategies when dealing with hectic examinations and to seek help when needed.

**Figure 1:**
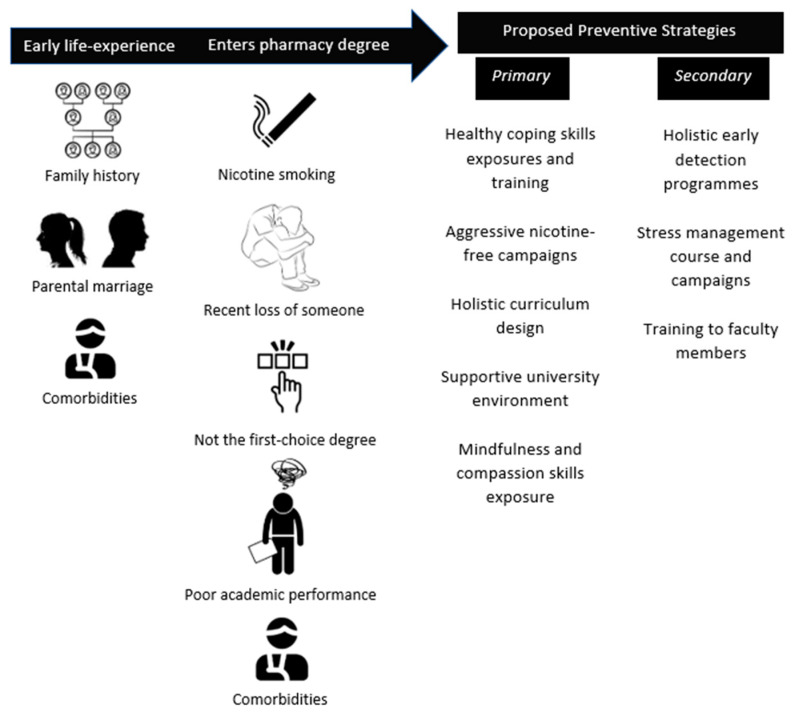
Factors associated with depression symptoms and proposed preventive strategies.

As for secondary preventive measures, early detection and management are advisable to prevent the complications associated with mental illnesses. This study found that students with early life experience through family history of mental illness, low family income, recent loss of someone close, underlying comorbidities and separated parents may be prone to developing depression syndrome. Family history is a predictor of mental illnesses in the offspring of parents with major depressive disorder [48]. Children who went through parental divorce may have faced a traumatic experience which had a negative impact on the child’s mental health [49]. Early secondary preventive program efforts made by the university to address a wide variety of concerns from academic to interpersonal in order to detect and wane early signs of depressive symptoms among at-risk students are recommended. The preventive programs that can be regularly undertaken at the university level include stress management courses and campaigns through yoga, breath work, meditation and mindfulness [50]. Faculty members can also be trained in these areas in addition to communication and counselling skills when handling students at risk of mental health issues. Figure 1 illustrates our findings and a summary of the proposed primary and secondary preventive strategies.

Our results showed that race and religion are important elements to consider for the mental well-being of university students. Similar findings from the 2019 National and Health Morbidity Survey (NHMS) reported that people of the Malay race and natives of Sabah and Sarawak had the highest prevalence of mental health problems [51]. Students brought up with a strong cultural or religious background often have influences on their daily life routine, including responses to stressors. Negative religious coping, such as feeling abandoned and punished by God, is significantly associated with depression symptoms [52]. In a country with strong multicultural and religious populations, careful planning of positive religious coping strategies can be made known regularly for university students as an alternative approach when experiencing stressful situations.

### Study Limitations

Firstly, due to the nature of this cross-sectional study through a web-based questionnaire where the response rate was likely to be low, a definitive conclusion cannot be made and generalized to all pharmacy students. If the response rate is more than 70%, the associations may be different. Previous studies have shown that surveys related to mental health issues may have low response rates [53,54]. Furthermore, students with existing clinical depression or low mood may be less motivated to respond to the survey. Secondly, all questionnaires are self-rated and therefore prone to recall bias. Thirdly, we did not proceed with a multivariable regression analysis because of the subjective nature of the interconnectedness between the independent variables, which may not be well captured and reflected correctly in the regression analysis. Instead, this study explored in detail the mean depression score in association with the investigated variables (Table 3) and binary logistic regression (Table 4) to complement the findings of the observed relationship differences seen in Table 2. With this, the stakeholders can consider the factors needed for developing primary and secondary preventive strategies for students’ well-being.

## 5. Conclusions

The main research results show that students with early life experience of family history of mental illness, separated parents and comorbidities were associated with depression symptoms. In addition, students who smoke nicotine, have recently lost someone close, who had a different first choice of degree and with poor academic performance were also associated with depression symptoms. The findings of this study suggest more resource allocation for targeted primary and secondary preventive strategies for managing mental health among university students. Strategies that develop healthy coping skills for stress management are important to introduce early in the first year of students’ university life in addition to nicotine-free campaigns. Universities can collaborate with health and social sectors to conduct simultaneous healthy coping skills programs and mindfulness and compassion skills activities to support students who are at high risk of developing mental illnesses. The carefully designed curriculum and its delivery must support and empower students towards a career pathway. Secondary preventive measures aiming for early detection of students with early life crisis experience with separated parents, family history of mental illness and underlying comorbidities should include a supportive university environment and training of faculty, which will benefit not only students but also families and university communities. Future research should investigate the impact of such interventions at the faculty and university levels on university students’ overall mental health and well-being.

## Figures and Tables

**Table 1:** Sociodemographic profile of participating undergraduate pharmacy students (n = 610).

Sociodemographic Profile		Frequency	Percentage (%)
**Age**	18–20 years old	78	12.8
	20–24 years old	512	83.9
	25–29 years old	20	3.3
**Gender**	Male	98	16.1
	Female	512	83.9
**Ethnicity**	Malay	500	82.0
	Chinese	61	10.0
	Indian	36	5.9
	Others	13	2.1
**Religion**	Islam	511	83.7
	Buddhism	48	7.9
	Hinduism	29	4.8
	Christianity	14	2.3
	Others	8	1.3
**Nicotine smoking**	Yes	8	1.3
	No	602	98.7
**Current place of residence**	Home	152	24.9
	Hostel	318	52.1
	Rental house outside campus	139	22.8
	Others	1	0.2
**Marital status**	Single	572	93.8
	Married	2	0.3
	Divorced	1	0.2
	In a serious relationship	35	5.7
**Year of study**	Year 1	127	20.8
	Year 2	149	24.4
	Year 3	143	23.4
	Year 4	191	31.4
**Study institution**	Public	367	60.2
	Private	243	39.8
Family monthly income *	<USD 482	132	21.6
	USD 482–1204	182	29.8
	USD 1204–1927	121	19.8
	>USD 1927	175	28.8

* USD 1 = MYR 4.15.

**Table 2:** Significant associations of sociodemographic, psychosocial and academic profiles of pharmacy students with depression symptoms (n = 610).

Profiles		Depression Symptoms		*X*^2^-Statistics (df)	*p* Value
		No	Yes		
**Sociodemographic**					
**Nicotine smoking**	Yes	1 (12.5%)	7 (87.5%)	-	0.03 ^b^
	No	320 (53.2%)	282 (46.8%)		
**Psychosocial**					
**Parental marital status**	Still together	292 (54.5%)	244 (45.5%)	6.09 (1)	0.01 ^a^
	Separated/divorced/ widowed	29 (39.2%)	45 (60.8%)		
**Recent loss of someone close within the past 1 year**	Yes	72 (45.0%)	88 (55.0%)	5.05 (1)	0.03 ^a^
	No	249 (55.3%)	201 (44.7%)		
**Academic matters**					
**Pharmacy degree as first choice to study**	Yes	217 (56.4%)	168 (43.6%)	5.85 (1)	0.02 ^a^
	No	104 (46.2%)	121 (53.8%)		
**cGPA**	2.00–2.99	40 (42.1%)	55 (57.9%)	8.87 (1)	0.01 ^a^
	3.00–3.49	156 (51.0%)	150 (49.0%)		
	3.50–4.00	125 (59.8%)	84 (40.2%)		
**Other matters**					
**Family history of mental illness**	Yes	21 (36.8%)	36 (63.2%)	6.28 (1)	0.01 ^a^
	No	300 (54.2%)	253 (45.8%)		
**Comorbidity**	Yes	61 (41.8%)	85 (58.2%)	9.05 (1)	0.003 ^a^
	No	260 (56.0%)	204 (44.0%)		

^a^ Pearson’s chi-square test. ^b^ Fisher’s exact test.

**Table 3:** Significant difference in mean depression score of DASS-42 between groups (n = 610).

Profiles		Mean Depression Score (±SD)	Mean Difference (95% CI)	t-Statistics (df)/F-Statistics (df1,df2)	*p*-Value
**Sociodemographic**					
**Age**	18–20 years old (n = 78)	8.58 (±7.52)	-	3.05 (2, 607)	0.05 ^b^
	20–24 years old (n = 512)	11.20 (±9.39)			
	25–29 years old (n = 20)	12.50 (±10.18)			
**Ethnicity**	Malay (n = 500)	11.22 (±9.30)	-	4.76 (3, 606)	0.003 ^b^
	Chinese (n = 61)	6.92 (±6.70)			
	Indian (n = 36)	12.19 (±10.74)			
	Others (n = 13)	13.92 (±8.27)			
**Religion**	Islam (n = 511)	11.26 (±9.27)	-	4.24 (4, 605)	0.001 ^b^
	Buddha (n = 48)	6.75 (±6.82)			
	Hindu (n = 29)	10.84 (±10.84)			
	Christian (n = 14)	5.93 (±5.51)			
	Others (n = 8)	15.63 (±9.94)			
**Nicotine smoking**	Yes (n = 8)	18.50 (±10.24)	7.7 (1.3–14.1)	2.35 (608)	0.02 ^a^
	No (n = 602)	10.80 (±9.18)			
Family income *	USD 482–1204 (n = 314)	11.22 (±8.97)	1.66 (0.2–3.1)	2.30 (608)	0.02 ^a^
	USD 1204–1927 (n = 296)	9.56 (±8.82)			
**Psychosocial**					
**Recreational drug use**	Regular user (n = 6)	20.50 (±14.85)	9.67 (2.2–17.1)	2.55 (608)	0.01 ^a^
	No (n = 604)	10.83 (±9.17)			
**Parental marital status**	Still together (n = 536)	10.53 (±8.98)	3.11 (0.9–5.3)	−2.73 (608)	0.02 ^a^
	Separated/divorced/ widowed (n = 74)	13.64 (±10.53)			
**Recent loss of someone close within the past 1 year**	Yes (n = 160)	12.53 (±9.68)	2.20 (0.5–3.9)	2.60 (608)	<0.01 ^a^
	No (n = 450)	10.33 (±9.01)			
**Academic matters**					
**Pharmacy degree as students’ first choice to study**	Yes (n = 385)	9.90 (±8.39)	2.72 (0.7–4.6)	−3.54 (608)	<0.001 ^a^
	No (n = 225)	12.62 (±10.31)			
**cGPA**	2.00–2.99 (n = 95)	13.58 (±10.28)	-	8.19 (2, 607)	<0.001 ^b^
	3.00–3.49 (n = 306)	11.27 (±9.49)			
	3.50–4.00 (n = 209)	9.15 (±7.95)			
**Other matters**					
**Family history of mental illness**	Yes (n = 57)	15.33 (±10.45)	4.88 (2.4–7.4)	3.84 (608)	<0.001 ^a^
	No (n = 553)	10.45 (±8.98)			
**Comorbidity**	Yes (n = 146)	10.16 (±8.88)	3.1 (1.3–4.9)	−3.37 (608)	<0.001 ^a^
	No (n = 464)	13.26 (±9.94)			

^a^ Independent T-test. ^b^ One-way ANOVA with post hoc analysis. * USD 1 = MYR 4.15.

**Table 4:** Binary logistic regression analysis predicting depression symptoms.

Independent Variables	Beta Coefficient	Level of Significance	Odd Ratio	95% Confidence Interval
**Nicotine smoking**	−2.01	0.67	0.13	0.016–1.15
**Parents’ marital status**	0.54	0.04 *	1.72	1.03–2.88
**Recent loss of someone close within the past year**	−0.34	0.08	0.72	0.49–1.04
**Pharmacy degree as students’ first choice to study**	0.31	0.79	1.36	0.96–1.93
**cGPA**	−0.55	0.04 *	0.57	0.34–0.95
**Family history of mental illness**	−0.74	0.01 *	0.48	0.27–0.85
**Comorbidities**	0.53	0.007 *	1.70	1.15–2.51

* Significant at *p* < 0.05.

## Supplementary Materials

The following supporting information can be downloaded at: https://www.mdpi.com/article/10.3390/ijerph19159629/s1, Table S1: Summary of Sociodemographic, Psychosocial and Academic Variables for Association Study; Table S2: Sociodemographic, Psychosocial and Academic Profiles of Pharmacy Students based on Depression Symptoms (n = 610); Table S3: Differences in mean depression scores based on DASS-42 between groups (n = 610).
