# Supplementary material for: Targeted Primary and Secondary Preventive Strategies for Depression among Malaysian Pharmacy Students
Source: Int J Environ Res Public Health. 2022 Aug 5;19(15):9629. doi: 10.3390/ijerph19159629 (PMC9367753; doi:10.3390/ijerph19159629)
Supplement: Supplementary file 1 [file ijerph-19-09629-s001.zip › ijerph-1768540-supplementary.pdf]

Supplementary Table S1. Summary of Sociodemographic, Psychosocial and Academic Variables for Association Study

Domain / variables	Measure / data item
Sociodemographic	
Age	Years
Gender	Female / Male
Ethnicity	Malay / Chinese / Indian / Others
Religion	Islam / Buddha / Hindu / Christian / Others
Nicotine smoking	Yes / No
Current place of residence	Home / Hostel / Rental house / Others
Marital status	Yes / No
Year of study	Year 1 / Year 2 / Year 3 / Year 4
Study institution	Public / Private
Family monthly income*	USD 482 – < USD 1204 / USD 1204 – USD 1927 / >USD 1927
Psychosocial	
Alcohol consumption	Yes / No
Recreational drug usage	Regular user / No
Parental marital status	Still together / Separated, Divorced
Recent loss of someone close within the past 1 year	Yes / No
Academic Matters	
Academic degree as first choice to study	Yes / No
Scholarship	Yes / No
CGPA	2.00 – 2.99 / 3.00 – 3.49 / 3.50 – 4.00
Other Matters	
Family history of mental illness	Yes / No
Comorbidities	Yes / No

*1 USD = RM4.15

Supplementary Table S2. Sociodemographic, Psychosocial and Academic Profiles of Pharmacy Students based on Depression Symptoms (n = 610)

Profiles		Depression Symptoms		X^2-statistics (df)	*p* value
		No	Yes		
Sociodemographic					
**Age**	< 20 years old	50 (64.1%)	28 (35.9%)	5.02 (1)	0.08[a]
	20 – 24 years old	262 (51.2%)	250 (48.8%)		
	25 – 29 years old	9 (45.0%)	11 (55.0%)		
**Gender**	Male	56 (57.1%)	42 (42.9%)	0.96 (1)	0.33[a]
	Female	265 (51.8%)	247 (48.2%)		
**Ethnicity**	Malay	257 (51.4%)	243 (48.6%)	1.66 (1)	0.20[a]
	Non-Malay	64 (58.2%)	46 (41.8%)		
**Religion**	Muslim	261 (51.1%)	250 (48.9%)	3.02 (1)	0.08[a]
	Non-Muslim	60 (60.6%)	39 (39.4%)		
**Nicotine smoking**	Yes	1 (12.5%)	7 (87.5%)	-	0.03[b]
	No	320 (53.2%)	282 (46.8%)		
**Place of residence**	Hostel	173 (54.4%)	145 (45.6%)	0.84 (1)	0.36[a]
	Non-hostel	148 (50.7%)	144 (49.3%)		
**Marital status**	Single	305 (53.3%)	267 (46.7%)	1.80 (1)	0.18[a]
	Non-single	16 (42.1%)	22 (57.9%)		
**Year of study**	Year 1	76 (59.8%)	51 (40.2%)	3.52 (1)	0.32[a]
	Year 2	75 (50.3%)	74 (49.7%)		
	Year 3	71 (49.7%)	72 (50.3%)		
	Year 4	99 (51.8%)	92 (48.2%)		
**Institution**	Public	195 (53.1%)	172 (46.9%)	0.09 (1)	0.80[a]
	Private	126 (51.9%)	117 (48.1%)		
**Family monthly income***	< USD1204	157 (50.0%)	157 (50.0%)	1.79 (1)	0.18[a]
	> USD1204	164 (55.4%)	132 (44.6%)		
Psychosocial					
**Alcohol consumption**	Yes	17 (43.6%)	22 (56.4%)	1.36 (1)	0.24[a]
	No	304 (53.2%)	267 (46.8%)		
**Recreational drug usage**	Yes	1 (16.7%)	5 (83.3%)	-	0.11[b]
	No	320 (53.0%)	284 (47.0%)		
**Parental marital status**	Still together	292 (54.5%)	244 (45.5%)	6.09 (1)	0.01[a]
	Separated/ divorced/ widowed	29 (39.2%)	45 (60.8%)		
**Recent loss of someone close**	Yes	72 (45.0%)	88 (55.0%)	5.05 (1)	0.03[a]
	No	249 (55.3%)	201 (44.7%)		

**within the past 1 year**					
Academic matters					
**Pharmacy degree as first choice to study**	Yes	217 (56.4%)	168 (43.6%)	5.85 (1)	0.02[a]
	No	104 (46.2%)	121 (53.8%)		
**Scholarship**	Yes	90 (51.7%)	84 (48.3%)	0.08 (1)	0.78[a]
	No	231 (53.0%)	205 (47.0%)		
**cGPA**	2.00 – 2.99	40 (42.1%)	55 (57.9%)	8.87 (1)	0.01[a]
	3.00 – 3.49	156 (51.0%)	150 (49.0%)		
	3.50 – 4.00	125 (59.8%)	84 (40.2%)		
Other matters					
**Family history of mental illness**	Yes	21 (36.8%)	36 (63.2%)	6.28 (1)	0.01[a]
	No	300 (54.2%)	253 (45.8%)		
**Comorbidities**	Yes	61 (41.8%)	85 (58.2%)	9.05 (1)	0.003[a]
	No	260 (56.0%)	204 (44.0%)		

[a] Pearson's Chi square test
[b] Fisher's Exact test
*1 USD = RM4.15

Supplementary Table S3. Differences in mean depression scores based on DASS-42 between groups (n=610)

**Profiles**		**Mean Depression Score (±SD)**	**Mean difference (95% CI)**	**t-statistics (df) / F-statistics (df1,df2)**	**p-value**
Sociodemographic					
**Age**	18 – 20 years old (n=78)	8.58 (±7.52)	-	3.05 (2, 607)	0.05[b]
	20 – 24 years old (n=512)	11.20 (±9.39)			
	25 – 29 years old (n=20)	12.50 (±10.18)			
**Gender**	Male (n=98)	10.37 (±9.46)	0.64 (-1.3 – 2.6)	-0.62 (608)	0.54[a]
	Female (n=512)	11.01 (±9.19)			
**Ethnicity**	Malay (n=500)	11.22 (±9.30)	-	4.76 (3, 606)	0.003[b]
	Chinese (n=61)	6.92 (±6.70)			
	Indian (n=36)	12.19 (±10.74)			
	Others (n=13)	13.92 (±8.27)			
**Religion**	Islam (n=511)	11.26 (±9.27)	-	4.24 (4, 605)	0.001[b]
	Buddha (n=48)	6.75 (±6.82)			
	Hindu (n=29)	10.84 (±10.84)			
	Christian (n=14)	5.93 (±5.51)			
	Others (n=8)	15.63 (±9.94)			
**Nicotine smoking**	Yes (n=8)	18.50 (±10.24)	7.7 (1.3 – 14.1)	2.35 (608)	0.02[a]
	No (n=602)	10.80 (±9.18)			
**Place of residence**	Home (n=152)	11.28 (±8.80)	-	1.34 (3, 606)	0.26[b]
	Hostel (n=318)	10.24 (±8.64)			
	Rental house outside campus (n=139)	11.99 (±10.83)			
	Others (n=1)	15.00 (±0.00)			
**Marital status**	Single (n=572)	10.83 (±9.25)	-	0.65 (3, 606)	0.58[b]
	Married (n=2)	10.50 (±9.20)			
	Divorced (n=1)	2.00 (±0.00)			
	In a serious relationship (n=35)	12.46 (±9.10)			
**Year of study**	Year 1 (n=127)	9.54 (±8.36)	-	1.24 (3, 606)	0.30[b]
	Year 2 (n=149)	11.36 (±9.45)			
	Year 3 (n=143)	11.48 (±8.70)			
	Year 4 (n=191)	11.03 (±9.95)			
**Institution**	Public (n=367)	10.51 (±8.64)	0.99 (-0.5 – 2.5)	-1.29 (608)	0.21[a]
	Private (n=243)	11.50 (±10.05)			
**Family income***	USD 482 – USD 1204 (n=314)	11.22 (±8.97)	1.66	2.30 (608)	0.02[a]

	USD 1204 – USD 1927 (n=296)	9.56 (±8.82)	(0.2 – 3.1)		
Psychosocial					
**Alcohol consumption**	Yes (n=39)	11.26 (±8.83)	0.38 (-2.6 – 3.4)	0.25 (608)	0.80[a]
	No (n=571)	10.88 (±9.27)			
**Recreational drug use**	Regular user (n=6)	20.50 (±14.85)	9.67 (2.2 – 17.1)	2.55 (608)	0.01[a]
	No (n=604)	10.83 (±9.17)			
**Parental marital status**	Still together (n=536)	10.53 (±8.98)	3.11 (0.9 – 5.3)	-2.73 (608)	0.02[a]
	Separated/ divorced/ Widowed (n=74)	13.64 (±10.53)			
**Recent loss of someone close within the past 1 year**	Yes (n=160)	12.53 (±9.68)	2.20 (0.5 – 3.9)	2.60 (608)	<0.01[a]
	No (n=450)	10.33 (±9.01)			
Academic matters					
**Pharmacy degree as students' first choice to study**	Yes (n=385)	9.90 (±8.39)	2.72 (0.7 – 4.6)	-3.54 (608)	<0.001[a]
	No (n=225)	12.62 (±10.31)			
**Scholarship**	Yes (n=174)	10.60 (±9.00)	0.43 (-1.2 – 2.1)	-0.52 (608)	0.60[a]
	No (436)	11.03 (±9.33)			
**cGPA**	2.00 – 2.99 (n=95)	13.58 (±10.28)	-	8.19 (2, 607)	<0.001[b]
	3.00 – 3.49 (n=306)	11.27 (±9.49)			
	3.50 – 4.00 (n=209)	9.15 (±7.95)			
Other matters					
**Family history of mental illness**	Yes (n=57)	15.33 (±10.45)	4.88 (2.4 – 7.4)	3.84 (608)	<0.001[a]
	No (n=553)	10.45 (±8.98)			
**Comorbidity**	Yes (n=146)	10.16 (±8.88)	3.1 (1.3 – 4.9)	-3.37 (608)	<0.001[a]
	No (n=464)	13.26 (±9.94)			

[a] Independent T-test

[b] One-way ANOVA with post hoc analysis

*1 USD = RM4.15
